# Influence of social determinants on lifestyle modification adherence in people with prediabetes: a sequential explanatory mixed-methods study protocol

**DOI:** 10.3389/fpubh.2025.1591205

**Published:** 2025-07-03

**Authors:** Ana Carvalho-Azevedo, Manuela Abbate, Sergio Fresneda, María Arias-Fernández, Marina Torres-Carballo, Aina M. Yañez, Cristina Moreno-Mulet, Miquel Bennasar-Veny

**Affiliations:** ^1^Department of Nursing and Physiotherapy, University of the Balearic Islands (UIB), Palma, Balearic Islands, Spain; ^2^Research Group on Global Health, University of the Balearic Islands (UIB), Palma, Balearic Islands, Spain; ^3^Research Group on Nursing, Community and Global Health, Health Research Institute of the Balearic Islands (IdISBa), Palma, Spain; ^4^Primary Care Research Unit of Mallorca, Public Health Service of the Balearic Islands (Ibsalut), Palma, Spain; ^5^Research Institute of Health Sciences (IUNICS), Palma, Spain; ^6^Research Network on Chronicity, Primary Care, and Health Promotion (RICAPPS), Institute of Carlos III, Madrid, Spain; ^7^Research Group on Qualitative and Critical Health (GICS), University of the Balearic Islands (UIB), Palma, Balearic Islands, Spain; ^8^Research Group on Care, Chronicity and Health Evidence (CurES), Health Research Institute of the Balearic Islands (IdISBa), Palma, Spain; ^9^CIBER de Epidemiología y Salud Pública (CIBERESP), Institute of Health Carlos III, Madrid, Spain

**Keywords:** therapeutic adherence and compliance, social determinants of health, prediabetic state, healthy lifestyle, social class, gender role

## Abstract

**Aim:**

Social determinants (SD) such as age, gender, ethnicity, postal code, or socioeconomic status, as well as health beliefs strongly impact health outcomes. This study aims to analyze the influence of SD on adherence to healthy lifestyle recommendations among individuals with prediabetes.

**Design:**

This sequential explanatory mixed-methods study will include an initial cross-sectional analysis of quantitative data, followed by a qualitative ethnomethodological study using critical discourse analysis.

**Methods:**

The quantitative analysis will use data from 103 participants with prediabetes included in the intervention arm of the PREDIPHONE trial. The relationship between adherence to the lifestyle modification intervention (diet and physical activity) and sociodemographic characteristics will be explored by multivariable linear regression. The qualitative study will explore how gender, social class, and other factors (such as social and family support, knowledge about one’s health condition, health beliefs, and patient-professional relationship) can influence adherence to lifestyle changes in a selected subgroup of individuals. Data generation techniques will include semi-structured interviews, discussion groups, support network mapping, and the researcher’s field diary. The rigor strategies that will be applied include triangulation, data saturation, and reflexivity.

**Discussion:**

Prediabetes exhibits an uneven distribution, disproportionately affecting individuals from underprivileged social classes, directly impacting on adherence behaviors. Our study can guide the development of health interventions tailored to individuals with prediabetes, focusing on addressing social disparities in lifestyle modification.

**Patient or public contribution:**

Participants will contribute through semi-structured interviews and discussion groups, providing insights into their experiences on adherence to lifestyle changes.

**Clinical trial registration:**

ClinicalTrials.gov, identifier NCT06488677.

## Background

Type 2 diabetes (T2D) is a major risk factor for adult morbidity and mortality and represents a public health burden due to its rising global prevalence (1). According to a recent systematic review, 529 million people worldwide suffered from T2D in 2021. This number is estimated to grow to a prevalence of 1.31 billion by 2050 (2). T2D is preceded by prediabetes, a phase characterized by higher-than-normal blood glucose levels that do not reach the threshold required for a T2D diagnosis (3). Currently, approximately 7.5% of the adult population is affected by prediabetes, and by 2030, more than 622 million people worldwide are projected to have this condition. It is estimated that 70% of people with prediabetes will eventually develop T2D, with a mean annual incidence rate ranging from 5 to 10% (4). Notably, the risk of developing T2D extends beyond physiological factors. Social determinants (SD) such as age, gender, ethnicity, postal code, or socioeconomic status, and health beliefs are strong predictors of disease onset and progression (1). Populations with a low socioeconomic status (i.e., economically disadvantaged backgrounds, lower health literacy levels, lower educational status, and often, from ethnic and racial minorities), are known to experience reduced access to healthcare including health promotion programs (5). They also have lower adoption rates of health behaviors, such as physical activity (PA) and healthy diet, which increases their risk of chronic diseases such as T2D and their likelihood to suffer inequalities in mortality (6).

Despite strong evidence that lifestyle interventions prevent T2D, individual adherence varies widely (7). To date, the majority of interventions targeting adherence have been limited to the pharmacological sphere and have often overlooked the complexity of the phenomenon. In the context of chronic diseases, it is essential to integrate social and contextual factors to develop personalized strategies to improve adherence and sustain healthy behaviors (8).

Since SD strongly influence adherence to healthy lifestyle changes, deficiencies in any of the SD can create significant barriers to self-care for individuals with T2D (9). Studies exploring factors that can potentially influence the adherence process cover individual aspects such as age, gender, health beliefs, the patient-professional relationship, and social factors such as socioeconomic status, support networks, social cohesion, and family support (10).

Age has been shown to be a determining factor on adherence to healthy lifestyles as older individuals demonstrate better adherence in the management of T2D than younger ones (11). Gender differences also influence self-care, with women showing higher adherence rates despite having less time available for PA compared to men (12). Health beliefs are also a vital element that can modulate self-care behaviors. In the case of prediabetes, the perception of the risk of progressing to T2D is generally low, which could limit adherence to positive lifestyle changes (13). The patient-professional relationship, including health communication, is another aspect associated with disease management. A lack of personal touch in communication can lead to superficial and distant interaction, which can negatively affect treatment adherence (14). Moreover, individuals with a high socioeconomic status adopt and maintain healthier behaviors more frequently than those with a low socioeconomic status (15). Social support and cohesion also emerge as relevant adherence behaviors. Support network has been associated with adherence behaviors and health promotion, while the lack of it has a negative effect on adherence (2). Finally, family support can significantly influence self-management, as patients with more family support have higher levels of self-care and compliance to pharmacological treatment (16).

Successful adherence to lifestyle modifications involves more than individual effort and responsibility; it requires understanding the interplay between sociodemographic variables and the psychosocial environment within the individual’s broader context (13).

For all of the above, this study aims to explore the influence of SD on adherence behaviors in individuals with prediabetes included in the PREDIPHONE trial, using a mixed-methods sequential explanatory design. The findings from both the quantitative and qualitative phases will be integrated to offer a comprehensive understanding of the complex phenomenon of adherence.

## Methods/design

### Purpose of the study

The main aim of this study is to analyze the influence of SD on adherence to healthy lifestyle recommendations in individuals with prediabetes included in the intervention arm of the PREDIPHONE trial. The project hypothesizes that social determinants of health (gender, age, education level, and socioeconomic status) influence the level of adherence to a healthy lifestyle intervention in these individuals. Additionally, the following secondary hypothesis are proposed:

1) Positive psychosocial factors, social environment, and patient-professional relationship may increase adherence to a healthy lifestyle intervention in people with prediabetes.
2) Individuals from socioeconomically disadvantaged backgrounds are likely to exhibit lower adherence to the intervention.

The study aims to achieve the following objectives:

(1) Evaluate whether SD, specifically gender, age, social class, and educational level, influence adherence to healthy lifestyle recommendations—including a healthy diet and regular PA—among individuals with prediabetes.
(2) Explore how perceived contextual and social factors—such as social and family support, knowledge about one’s health condition, health beliefs, and the patient-professional relationship—can act as facilitators or barriers to adherence to lifestyle changes in these individuals.

To guide the integration of the quantitative and qualitative findings, the following mixed-methods research question is proposed: *How will the relationship between sociodemographic variables—namely age, sex, educational level and socioeconomic status—and levels of adherence to a lifestyle modification intervention help explain contextual and interpersonal factors contributing to adherence to healthy lifestyle recommendations among individuals with prediabetes?*

### Design

This mixed-methods study is conducted with a sequential explanatory design, integrating quantitative analysis with an ethno-methodological qualitative study. The first phase involves a quantitative cross-sectional analysis of data from the PREDIPHONE trial. The results of this quantitative phase will inform the subsequent qualitative phase. The qualitative phase will employ an ethnomethodological framework along with a critical discourse analysis (CDA) approach. Ethnomethodology, as an interpretative sociological perspective, focuses on understanding how individuals attribute meaning to their social interactions and daily practices (17). On the other hand, CDA serves as a methodological tool that critically examines speech, text, and language to uncover the sociocultural and political realities of specific social contexts underlying discourse constructs. These approaches will be used in a complementary way: ethnomethodology will guide the interpretation of experiences and meanings constructed in everyday interactions, while CDA will examine how these meanings are shaped by broader social, cultural, and power structures, also serving as a tool for social critique and transformation (18). Finally, methodological integration will involve connecting participants across phases, building on quantitative findings, and weaving together qualitative and quantitative results to identify convergences and divergences.

**Figure 1:**
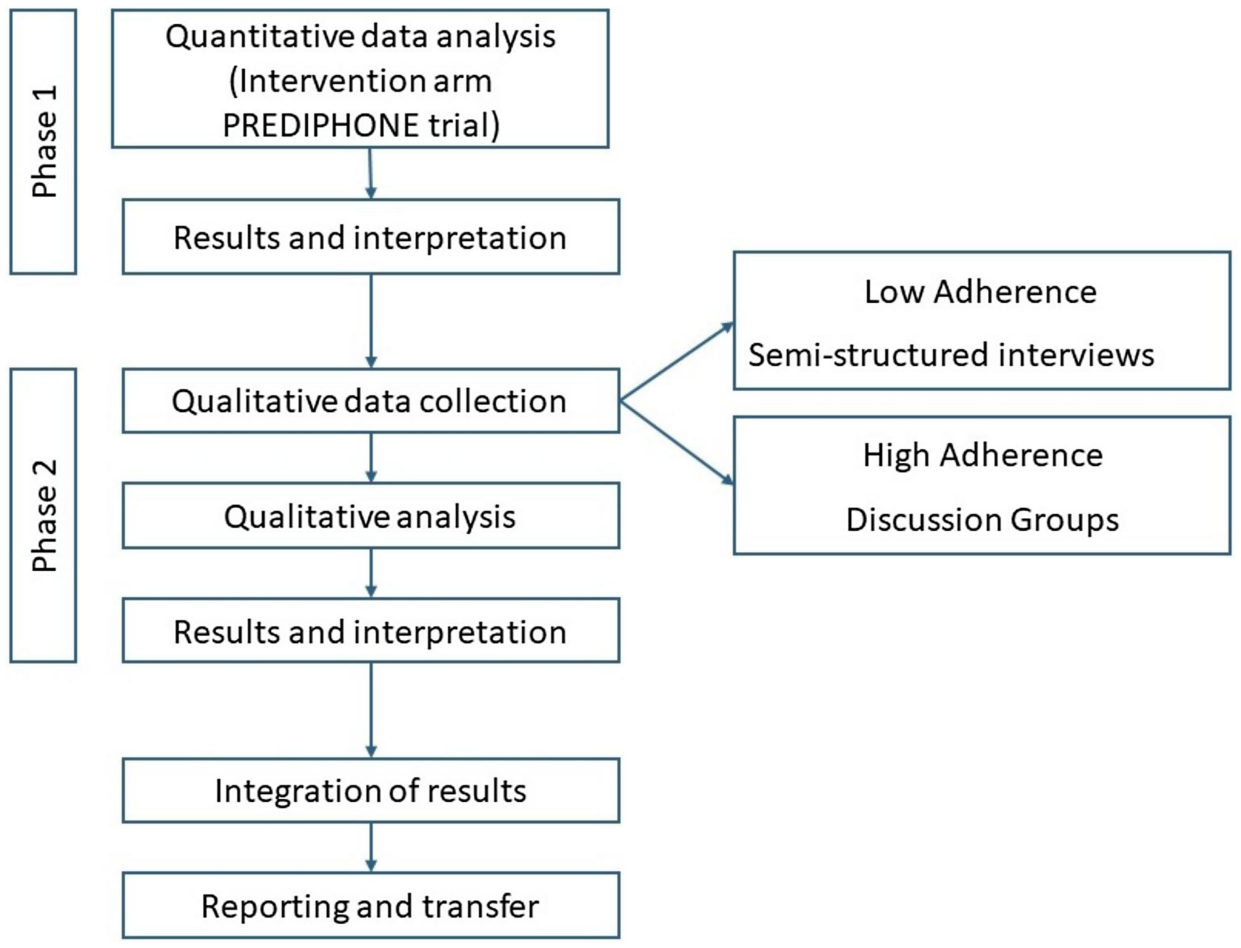
Study flow-diagram.

The adoption of this design can facilitate a deeper understanding of the complexity surrounding adherence behaviors. The two phases of this study are outlined in Figure 1.

### Quantitative phase (Phase 1)

The quantitative phase will consist of a cross-sectional analysis of data from the intervention arm of the PREDIPHONE trial (19). Data from 103 participants randomized to the intervention arm will be used to analyze the relationship between sociodemographic variables and adherence to the lifestyle intervention (diet and PA). Baseline data will be used to describe the sample.

#### The PREDIPHONE trial

The study design and methods of the PREDIPHONE trial have been previously described (19). Briefly, the PREDIPHONE trial is a randomized controlled trial aimed at evaluating the effectiveness of a nurse-led personalized telephone lifestyle intervention in reducing fasting plasma glucose (FPG) in adults with prediabetes, compared to automated text messages (SMSs). Between May 2021 and September 2022, a total of 206 participants were screened and recruited in 5 primary care centers of Palma, Mallorca.

Eligibility criteria included: age between 20 and 75 years, FPG between 100 y 125 mg/dL; body mass index (BMI) between ≥27 and <40 kg/m^2^; and written informed consent. Further details of the inclusion/exclusion criteria can be found in the published protocol.

Randomization was performed using permuted blocks of 4 in a 1:1 ratio via an internet-based program (OxMaR). The allocation was concealed to all staff members involved in the trial.

Participants allocated to the intervention group received personalized dietary and PA advice throughout the 9-month intervention period. At baseline, participants received supporting information material and agreed upon behavioral goals and action plans with the nurse. Nurses delivered between 8 and 12 individual telephone consultations to provide dietary and PA advice and assess compliance to recommendations. The consultations were structured according to the 5A’s behavior change model (Assess, Advise, Agree, Assist, Arrange), encouraging participants engagement in self-management. Goals and action plans were individualized based on baseline behavior and preferences.

Dietary advice followed a four-step approach: promoting Mediterranean dietary choices, using the Healthy Eating Plate model, educating on portion sizes, and, when appropriate, advising on caloric restriction. Participants were also encouraged to reduce salt, stay hydrated, and read food labels. Physical activity advice followed the 2020 World Health Organization (WHO) guidelines, recommending 150–300 min of moderate or 75–150 min of vigorous aerobic activity per week, along with strength training and reduced sedentary time. A five-step model was used to gradually increase frequency from one to three days per week of both exercise types. Participants were also encouraged to include stretching after each session.

Participants in the control group received a total of 150 automated SMSs (160 characters) on lifestyle advice throughout the 9-month intervention period.

Study data were collected at baseline, at months 4 and 9 of the intervention period, and at month 15 (post-intervention).

### Qualitative phase (Phase 2)

#### Theoretical framework

This study will be conducted within the critical-social paradigm, focusing on how SD (specifically gender and social class) can influence health behaviors and, in turn, generate health inequalities or inequities. The critical-social paradigm emerged as a tool to understand societal conditions as a basis for social transformation. In terms of health, by considering more deeply the sociocultural context of the individuals, nurses can work to empower them to adopt a critical perspective on their health, contributing to reduce health inequalities or inequities (20).

The WHO’s Conceptual Framework for Action on the Social Determinants of Health will be used to understand and describe the relationship between SD and health (21). Specifically, the influence of social class on adherence will be explored using the perspectives of Marx, Weber, and Wright (22). Social class is a fundamental factor that influences how relationships and processes develop, thereby creating and perpetuating significant inequalities in society. These deeply rooted inequalities are important to consider when analyzing why individuals adhere or do not adhere to healthy behaviors. Essentially, social class affects various aspects of life, including access to resources and health opportunities, which can impact one’s ability to maintain healthy habits.

The influence of gender on adherence behaviors will be addressed through two theories: Nancy Fraser’s three-dimensional theory and Carme Valls’ middle-range theory on gender and health. Nancy Fraser’s perspective will contribute to exploring how gender-related injustices are reflected in the health domain (23). Carme Valls’ theory can contribute to understand how gender inequalities intersect with other forms of oppression, such as social class and race, impacting a variety of health phenomena (24).

The patient-professional relationship will be analyzed using Jürgen Habermas’ Theory of Communicative Action (TCA) (25) and Paulo Freire’s Learning Theory, or more specifically, his concept of Popular Health Education (26). These theories can be applied to the health domain to help understand how the patient-professional communication can assist in improving self-care practices.

In the health context, TCA can help examining how nursing communication can empower and influence health behaviors both rationally and humanely. Communication in health should strengthen trust, lead to autonomy, and provide support throughout the process, respecting the patients’ rights to speak and be heard (27). The Paulo Freire’s Learning Theory and his concept of Popular Health Education can be applied to explore the role of nurses in acknowledging and respecting individual knowledge to eventually transform reality through autonomy and fostering the development of critical individuals who are aware of their own needs (26).

## Participants

### Quantitative phase

The quantitative phase will include 103 participants of the PREDIPHONE trial assigned to the intervention group who completed the 9-month intervention period. Participants from the control group or those who withdrew from the trial will be excluded. With a sample of 103 subjects, there is a statistical power greater than 90% to detect correlations ranging from 0.4 to 0.7, assuming a significance level below 0.05.

### Qualitative phase

Inclusion criteria for the qualitative phase are: (1) participants randomized to the intervention group of the PREDIPHONE trial who completed the 9-month intervention period; (2) who voluntarily agree to participate in the study and sign the informed consent. Exclusion criteria are: (1) participants randomized to the control group or who dropped out of the PREDIPHONE trial; (2) who are not qualified for interviews or discussion groups (in the case of the latter, having a parental or work relationship with another member of the group).

Eligible participants will be intentionally sampled according to their level of adherence to the intervention (low/high), gender, and social class (white collar/blue collar). They will be contacted by phone and invited to participate. Between 12 and 16 participants with low adherence will be invited for individual semi-structured interviews. Between 18 and 24 participants with high adherence will be invited to participate in discussion groups (3/4 groups of 6/8 participants each). Both interviews and discussion groups will ensure a balanced representation of social class and gender. If possible, white- and blue-collar participants will be allocated in separate discussion groups to avoid potential inhibition and ensure that each group feels comfortable sharing their unique perspectives and experiences. The final participant sample will be determined according to the “information power” criteria, which assesses the participants’ ability to provide rich and relevant data by considering the following factors: (a) the study objectives, (b) the specificity of the sample, (c) the application of established theory, (d) the quality of dialogue, and (e) the analytical strategy employed (28).

Tables 1 and 2 present the initial participant sample categorized by profiles for semi-structured interviews and discussion groups.

**Table 1:** Profiles of participants for semi-structured interviews (low adherence).

**Characteristics of participants for semi-structured interviews**	**Number of participants**
Gender	
Female	6–8
Male	6–8
Social class	
Blue collar	
*Female*	3–4
*Male*	3–4
White collar	
*Female*	3–4
*Male*	3–4
**Total number of interviews**	**12–16**

**Table 2:** Profiles of participants for discussion groups (high adherence).

**Characteristics of participants for discussion groups**	**Number of participants**
Gender	
Female	12-16
Male	6–8
Social class	
Blue collar	
*Female*	6-8
*Male*	3–4
White collar	
*Female*	6-8
*Male*	3–4
**Total number of participants**	**18–24**

## Data collection and analysis

### Quantitative phase

Adherence to the intervention will be measured through the generation of a composite index divided into quartiles. Participants scoring below the 25th quartile will be classified as “low adherent,” while those scoring above the 75th will be classified as “high adherent.”

Dietary adherence will be assessed using the 14-item PREDIMED Mediterranean Diet questionnaire (29); PA adherence will be evaluated using the REGICOR Abbreviated Questionnaire on PA in leisure time (30). The results from the dietary and PA questionnaires will be standardized and converted into z-scores to achieve a standard deviation of 1 and a mean of 0. These scores will be combined into a single value, which will be divided into quartiles of adherence.

Sociodemographic data (age, gender, educational level, and social class) will be collected during the baseline visit, in accordance with the study protocol. According to the occupation declared, subjects will be categorized as white or blue collar (22).

#### Data analysis

The main dependent variable will be the level of adherence to the recommendations. Statistical analysis will be performed using the SPSS version 26.0 statistical package (IBM, New York, USA). A descriptive analysis of the variables will be conducted to identify outliers. Frequencies and percentages will be used to describe each qualitative variable. To examine the association between adherence to a healthy lifestyle and sociodemographic variables (age, sex, social class, education level), a linear regression model adjusted for potential confounders will be used. The significance level will be set at *p* < 0.05.

### Qualitative phase

#### Data generation techniques

Data will be collected via semi-structured interviews and discussion groups.

#### Semi-structured interviews

Semi-structured interviews will be conducted individually with participants showing low adherence to the intervention. This method provides a comfortable and private setting that encourages participants to express themselves freely, without the potential pressure of peer judgment. The interviews will be conducted at the participant’s reference Primary Healthcare Center to ensure confidentiality and comfort. Each interview is expected to last between 40 and 60 min. Table 3 provides preliminary scripts that will serve as guides for the semi-structured interviews. These scripts can be adjusted to better align with the specific characteristics of the participants involved.

**Table 3:** Preliminary scripts for the semi-structured interviews and discussion groups.

Preliminary script for the semi-structured interview
What did you know about diabetes before entering this program? What changes in lifestyle have you made so far?
How did you manage your diet? What resources did you have available?
Did you engage in regular exercise? Could you elaborate on your routine? What resources did you have available?
Which aspects of a healthy routine (healthy diet and regular exercise) were easier or more difficult for you to follow? Why?
How would you describe your relationship with the healthcare professionals regarding the management of prediabetes?

Preliminary script for the Discussion groups
How was the experience of participating in the PREDIPHONE trial?
Which aspects were easier for you to comply with? Why?
Which aspects were more complicated to follow? Why?
How was your relationship with the professionals?

#### Discussion groups

The discussion groups will be used for those participants who show high adherence to the intervention. This format allows participants to share their successful experiences and strategies in adopting lifestyle changes related to diet and PA with others, thereby promoting a collaborative environment.

The groups will meet at the University of the Balearic Islands, on dates and times convenient for the participants. To ensure comfort during discussions, each group will be made up of 6 individuals who have not had any prior relationship with each other. Sessions will last 60–120 min, and the facilitator will guide the discussion topics. This duration is set to allow sufficient time for thorough discussion without causing verbal fatigue (31). During the sessions, participants will be invited to share a small snack, which will also serve to explore their dietary practices and facilitate conversation.

Both the semi-structured interviews and the discussion groups will be conducted by an experienced interviewer with expertise in the phenomenon under investigation. Prior participant’s consent, the interviews will be audio-recorded to be later literally transcribed and analyzed. Participants in the discussion groups will also commit to maintaining strict confidentiality regarding the information shared. Table 3 provides preliminary scripts that could serve as a guide for the discussion groups. These scripts can be adjusted to better align with the specific characteristics of the participants involved.

#### Field diary

A field diary will be maintained throughout the entire interview process for the interviewer to document reflections and observations, including descriptive, methodological, and theoretical notes that will be essential for later interpretation and analysis (32).

#### Support network mapping

Before concluding the interviews or group sessions, participants will be asked to map their personal support network (33). The mapping will help the interviewer to understand the breadth and depth of the individual’s social network and support mechanisms and provide insights into how these factors might directly influence adherence behaviors.

#### Data analysis

The study will employ content and CDA as the primary analytical framework. CDA examines the complex relationship between language, power, and society, aiming to reveal and challenge how discourse shapes and is shaped by social structures. In this case, this analytical approach will focus on understanding how social inequalities influence adherence to lifestyle modifications (18). Data from the interviews and discussion groups will be coded and categorized using an inductive-deductive analysis (abductive analysis). The coding and categorization processes, along with data analysis, will be systematized and optimized using ATLAS.ti 23 software.

To ensure methodological rigor and validity, data will be collected until reaching saturation. Additionally, data triangulation will be employed to enhance the validity and comprehension of the study findings. The interviewer’s reflexivity will be critically assessed throughout the study, particularly during the data collection and analysis phases, through the researcher’s field diary.

### Data integration and interpretation

Quantitative findings (Phase 1) will inform the qualitative phase (Phase 2), specifically by guiding the recruitment of participants with low and high adherence to the intervention, based on social class and gender. This purposive sampling strategy will allow for a deeper and more contextualized understanding of the phenomenon under study. Methodological integration will be achieved through two main strategies: (1) “connecting” participants across all phases, and (2) “building” upon the quantitative results to address explanatory gaps identified in the data. Additionally, a “weaving approach” will be employed during the interpretation phase, enabling side-by-side integration of quantitative and qualitative results to highlight areas of convergence, complementarity or divergence (34).

To ensure inferential consistency, conclusions drawn from each strand will be examined for coherence and mutual reinforcement. Triangulation and framework-guided analysis will be used to strengthen the validity of the meta-inferences and to support integration across different paradigmatic perspectives (35).

### Ethical approval and consent to participate

This study was approved by the Research Ethics Committee of the Balearic Islands (CEI-IB: IB ref. 3947/19 PI). All researchers involved will sign a confidentiality agreement. Prior to initiating any study procedures, written informed consent will be obtained from all participants. The study will adhere to the recommendations outlined in the Declaration of Helsinki and in the Organic Law 3/2018 on Data Protection. To preserve confidentiality and anonymity, each participant will receive a code that will be used instead of their real names throughout the study, in both the quantitative and qualitative phases. Only researchers directly involved in the study will have access to these codes. In the qualitative phase, all necessary measures will be taken to ensure that the information provided by participants is treated confidentially.

The trial protocol is registered with ClinicalTrials.gov under protocol registration number NCT06488677.

### Validity and reliability

The present protocol was developed using the Good Reporting of a Mixed Methods Study (GRAMMS) guidelines (36). The quantitative results will be reported following the Observational studies in Epidemiology (STROBE) guidelines (37). The qualitative components will be reported following the Consolidated Criteria for Reporting Qualitative Research (38).

## Discussion

This study protocol outlines a mixed-method research project aimed at examining the influence of SD on adherence to recommended healthy lifestyles behaviors in individuals with prediabetes. Diabetes represents a considerable burden to public health worldwide due to its high prevalence and associated complications. Moreover, both prediabetes and diabetes exhibit an unequal distribution, disproportionately affecting individuals from underprivileged social classes (2). These health inequalities can also influence access and adherence to healthy lifestyle changes, making it essential to approach these conditions from a holistic perspective that considers not only the biology of the disease but also social and behavioral factors of the individual (13).

In this sense, this research could help comprehend the mechanisms by which social factors influence some individuals more than others to easily adhere to diet and regular PA. We also intend to identify which specific factors have a greater impact on the phenomenon of adherence.

Finally, this research could offer significant insights that can guide the development and implementation of tailored health programs targeting vulnerable populations, emphasizing social justice in access to health resources. Specifically, these interventions could provide guidance and support for people to independently manage their health and enhance their quality of life.

## Limitations and strengths

Studying adherence to lifestyle modifications in relation to SD involves addressing a multifactorial and inherently complex phenomenon, which can be difficult to fully capture and interpret. One anticipated limitation is the potential reluctance of participants with low adherence to engage in the qualitative phase, as they may be less inclined to share personal experiences or less motivated to participate. Another possible limitation concerns the participation of individuals from lower social class, who may face time constraints or limited availability due to demanding work schedules, which could hinder their ability to attend the interviews. A key strength of this study lies in its adoption of a pragmatic mixed-methods approach, integrating quantitative and qualitative methodologies. This design enables each method to contribute uniquely to the understanding of the research problem. The integration of both phases is structured to ensure consistency and coherence during interpretation. This approach is essential to explore adherence behaviors from multiple perspectives and to achieve a broader and deeper understanding of the phenomenon.
